# Cytochrome P450 1A1 genetic polymorphisms and risk of hepatocellular carcinoma among chronic hepatitis B carriers

**DOI:** 10.1038/sj.bjc.6690397

**Published:** 1999-05-01

**Authors:** M-W Yu, Y-H Chiu, S-Y Yang, R M Santella, H-D Chern, Y-F Liaw, C-J Chen

**Affiliations:** School of Public Health, andGraduate Institute of Epidemiology, College of Public Health, National Taiwan University, Taipei, Taiwan; Division of Environmental Health Sciences, School of Public Health, Columbia University, New York, NY 10032, USA; Graduate Institute of Pharmaceutical Sciences, College of Medicine, National Taiwan University, Taipei, Taiwan; Liver Research Unit, Chang-Gung Memorial Hospital and Chang-Gung MedicalCollege, Taipei, Taiwan

**Keywords:** chronic hepatitis B virus carriers, cigarette smoking, cytochrome P450 1A1, glutathione S-transferase M1, hepatocellular carcinoma, nested case-control study

## Abstract

Cigarette smoking has been associated with increased risk of hepatocellular carcinoma (HCC) in some epidemiological studies. Cytochrome P450 1A1 (CYP1A1) is involved in the biotransformation of tobacco-derived polycyclic aromatic hydrocarbons (PAHs) into carcinogenic metabolites. The aim of this study was to determine whether CYP1A1 polymorphisms were related to HCC risk among chronic hepatitis B virus (HBV) carriers. Genotypic variants of CYP1A1 were determined using polymerase chain reaction in 81 incident cases of HCC and 409 controls nested in a cohort study of 4841 male chronic HBV carriers. No overall association between CYP1A1 genotypes and HCC was observed. The presence of the MspI (odds ratio (OR) 3.15, *P*= 0.0196) or Ile-Val (OR 1.99, *P*= 0.0855) variant allele of CYP1A1 increased HCC risk among smokers, but posed no increased risk among non-smokers. The smoking-related HCC risk was most pronounced among those who had a susceptible allele of the CYP1A1 and a deficient genotype of glutathione S-transferase M1, which detoxifies PAH electrophilic metabolites produced by CYP1A1. In the absence of the Ile-Val variant allele, the MspI polymorphism was still associated with smoking-related HCC. This study suggests that tobacco-derived PAHs play a role in HCC risk among chronic HBV carriers, and CYP1A1 polymorphism is an important modulator of the hepatocarcinogenic effect of PAHs. The MspI and Ile-Val polymorphisms of CYP1A1 may have different mechanisms for increasing susceptibility to smoking-related HCC. © 1999 Cancer Research Campaign


*British Journal of Cancer* (1999) **80**(3/4), 598–603
© 1999 Cancer Research Campaign
Article no. bjoc.1998.0397

### Cytochrome P450 1A1 genetic polymorphisms and risk of hepatocellular carcinoma among chronic hepatitis B carriers

**M-W Yu[1,2], Y-H Chiu[2], S-Y Yang[2], RM Santella[3], H-D Chern[4], Y-F Liaw[5] and C-J Chen[2]**

[1]School of Public Health and [2]Graduate Institute of Epidemiology, College of Public Health, National Taiwan University, Taipei, Taiwan; [3]Division of Environmental Health Sciences, School of Public Health, Columbia University, New York, NY 10032, USA; [4]Graduate Institute of Pharmaceutical Sciences, College of Medicine, National Taiwan University, Taipei, Taiwan; [5]Liver Research Unit, Chang-Gung Memorial Hospital and Chang-Gung Medical College, Taipei, Taiwan

**Summary** Cigarette smoking has been associated with increased risk of hepatocellular carcinoma (HCC) in some epidemiological studies. Cytochrome P450 1A1 (CYP1A1) is involved in the biotransformation of tobacco-derived polycyclic aromatic hydrocarbons (PAHs) into carcinogenic metabolites. The aim of this study was to determine whether CYP1A1 polymorphisms were related to HCC risk among chronic hepatitis B virus (HBV) carriers. Genotypic variants of CYP1A1 were determined using polymerase chain reaction in 81 incident cases of HCC and 409 controls nested in a cohort study of 4841 male chronic HBV carriers. No overall association between CYP1A1 genotypes and HCC was observed. The presence of the MspI (odds ratio (OR) 3.15, $P = 0.0196$) or Ile-Val (OR 1.99, $P = 0.0855$) variant allele of CYP1A1 increased HCC risk among smokers, but posed no increased risk among non-smokers. The smoking-related HCC risk was most pronounced among those who had a susceptible allele of the CYP1A1 and a deficient genotype of glutathione S-transferase M1, which detoxifies PAH electrophilic metabolites produced by CYP1A1. In the absence of the Ile-Val variant allele, the MspI polymorphism was still associated with smoking-related HCC. This study suggests that tobacco-derived PAHs play a role in HCC risk among chronic HBV carriers, and CYP1A1 polymorphism is an important modulator of the hepatocarcinogenic effect of PAHs. The MspI and Ile-Val polymorphisms of CYP1A1 may have different mechanisms for increasing susceptibility to smoking-related HCC.

**Keywords:** chronic hepatitis B virus carriers; cigarette smoking; cytochrome P450 1A1; glutathione S-transferase M1; hepatocellular carcinoma; nested case-control study

Hepatocellular carcinoma (HCC) is a relatively rare tumour in the USA and Europe, but it is common in much of Asia and sub-Saharan Africa (Yu and Chen, 1994). There are almost 300 000 000 chronic hepatitis B virus (HBV) carriers in the world, with the highest prevalence in Southeast Asia, sub-Saharan Africa and Greenland (Tiollais et al, 1985). Although chronic HBV infection is the major cause of at least 80% of HCC throughout the world (Yu and Chen, 1994), only a minority of chronic HBV carriers are expected to develop HCC in their lifetime (Beasley, 1988). The fact that HCC is not an inevitable consequence of chronic HBV infection has stimulated the search for other risk factors. In addition to HBV, many other environmental factors have been documented as risk factors for HCC, including chronic infection with hepatitis C virus (HCV), aflatoxin exposure, cigarette smoking, alcohol drinking and low vegetable intake (Tsukuma et al, 1990; Chen et al, 1991, 1996; Yu et al, 1991; Yu et al, 1991, 1995*b*, 1997;Yu and Chen, 1994). Among environmental risk factors of HCC other than HBV, cigarette smoking is the most prevalent in the general population in Taiwan. However, the relative risk of HCC associated with cigarette smoking is low in the absence of chronic HBV or HCV infection (Chen et al, 1991; Yu et al, 1991). While there is convincing epidemiological evidence and widespread awareness of the role of cigarette smoke as a cause for cancer of the lung, upper aerodigestive tract and bladder (International Agency for Research on Cancer, 1986; Spitz et al, 1989; Talaska et al, 1991; Nakachi et al, 1993; Wynder and Hoffmann, 1994; Muscat et al, 1996), the root mechanism responsible for the action of cigarette smoking in hepatocarcinogenesis is not yet well understood.

Carcinogenic compounds in cigarette smoke which have been extensively reviewed include polycyclic aromatic hydrocarbons (PAHs), *N*-nitrosamines and aromatic amines (International Agency for Research on Cancer, 1986). The organospecificity of these tobacco-related carcinogens for the liver in humans is unknown. Most genotoxic chemical carcinogens are not intrinsically reactive but require metabolic conversion to DNA-binding intermediates (Guengerich and Shimada, 1991). Our recent study showing a significant association between genetic polymorphism in cytochrome P450 (CYP) 2E1, a critical enzyme in the metabolic activation of various low-molecular-weight procarcinogens, including tobacco-specific *N*-nitrosamines, and HCC among cigarette smokers suggests that *N*-nitrosamines may be involved in the aetiology of HCC (Yu et al, 1995*a*). Activated metabolites of carcinogens are subjected to metabolic conjugation and other kinds of detoxification. *N*-acetyltransferase 2 is a non-inducible

*Received 2 July 1998*
*Revised 1 October 1998*
*Accepted 20 October 1998*

*Correspondence to:* C-J Chen, Graduate Institute of Epidemiology, College of Public Health, National Taiwan University, No. 1 Jen-Ai Rd. Sec. 1, Room 1547, Taipei 100, Taiwan liver enzyme that deactivates carcinogenic aromatic amines via *N*-acetylation (Hein et al, 1993). Slow acetylators expressing little or no *N*-acetyltransferase 2 enzyme activity are at increased risk of HCC (Agundez et al, 1996).

None of the PAHs consistently found in cigarette smoke have been shown to be hepatocarcinogens in laboratory animals. No studies have been carried out to investigate the association between PAHs and HCC in humans. CYP1A1 is involved in the biotransformation of tobacco-derived PAHs such as benzo(a)pyrene (B(a)P) into reactive diol epoxide metabolites which can form covalent adducts with DNA (Shimada et al, 1992). Activated metabolites of B(a)P are subjected in part to metabolic detoxification by glutathione S-transferase M1 (GSTM1) (Mannervik and Danielson, 1988). The metabolic balance between activation and detoxification determines the biologically effective dose of carcinogens, thereby substantially influencing cancer risk. Two separate point mutations in the CYP1A1 gene have been reported: one within exon 7 that causes an isoleucine to valine substitution in the haem-binding region (Ile-Val polymorphism), and the other in the 3′ non-coding region (MspI polymorphism). The two polymorphisms are more frequent in Japanese than in Caucasians (Nakachi et al, 1993; Sivaraman et al, 1994). Cigarette smoking Japanese homozygous for the variant genotype of the MspI or Ile-Val polymorphisms who also had GSTM1 gene deletion, were found to be at remarkably high risk of lung cancer (Nakachi et al, 1993).

CYP1A1 was generally considered to be involved in extrahepatic carcinogenesis because early studies showed that the expression of CYP1A1 was low in human liver (Guengerich et al, 1991). However, recent studies using more sensitive techniques for the detection of CYP1A1 messenger RNA have demonstrated that CYP1A1 is expressed in a high proportion of human liver tissues (McKinnon et al, 1991; Schweikl et al, 1993). The role of CYP1A1 genetic polymorphism in susceptibility to HCC has not yet been investigated. This case-control study was conducted within a large-scale cohort of men in Taiwan to test the relationship between susceptibility to smoking-related HCC and genetic polymorphisms in CYP1A1. The gene–gene interaction between CYP1A1 and GSTM1 in the development of smoking-related HCC was also investigated.

### SUBJECTS AND METHODS

A cohort of 4841 male chronic carriers of HBV surface antigen (HBsAg) and 2501 non-carriers aged 30–65 years who were free of HCC was recruited from the Government Employee Central Clinics and the Liver Unit of Chang-Gung Memorial Hospital in Taiwan from August 1988 to June 1992. At recruitment, each study subject was personally interviewed to obtain information on sociodemographic characteristics (e.g. age and ethnicity), habits of smoking and alcohol drinking, dietary consumption, as well as a history of major chronic diseases. Each study subject was asked about the ethnicity of both parents. Data on chronic liver disease, including chronic hepatitis and liver cirrhosis, diagnosed by physicians were thus obtained. The diagnosis of chronic hepatitis in Taiwan was mainly based on sustained elevated serum aminotransferase levels for ≥ 6 months. Only a small fraction of the patients with chronic hepatitis were diagnosed histologically. Liver cirrhosis was diagnosed by clinical manifestation (including coagulopathy, hypoalbuminemia, abnormal liver function tests, haematologic evidence of hypersplenism, ascites, jaundice and portal hypertension with or without variceal bleeding), histological findings of liver biopsy and/or ultrasonography. Questionnaire interview was carried out by well-trained research assistants. Blood specimens, including serum and white blood cells, from the study subjects were also obtained and frozen at –70°C until subsequent analysis. All study subjects gave verbal or written informed consent for both the interview and blood collection.

Follow-up of the study subjects was performed through various channels: annual α-fetoprotein measurement and ultrasonography examination, personal telephone interview, and data linkage with computer files of national death certification and cancer registry systems. The diagnosis of HCC was based on (1) positive findings on cytological or pathological examination and/or (2) positive images on angiogram, ultrasonography and/or computed tomography, combined with an α-fetoprotein level ≥ 400 ng/ml. There were 92 incident cases of HCC identified during the follow-up period from August 1988 to June 1997. A total of 624 controls were randomly selected from cohort subjects who were not affected with HCC through the follow-up period. They were matched with cases on age (± 5 years), date of blood collection and questionnaire interview (± 3 months). Successful genotyping was obtained for 84 of the 92 case subjects and 563 (154 HBsAg-negative and 409 HBsAg-positive) of the 624 control subjects. Ninety-six percent (81/84) of the HCC cases with available data on CYP1A1 and GSTM1 genotypes were positive for HBsAg.

Genomic DNAs were isolated from peripheral leucocytes. For analysis of the MspI restriction fragment length polymorphism in CYP1A1 the following two primers were used: 5′-TAGGAGTCTTGTCTCATGCCT-3′ and 5′-CAGTGAAGAGGTGTAGCCGCT-3′ (Nakachi et al, 1993). Amplification was performed using initial denaturation at 94°C for 5 min followed by 30 cycles of 94°C for 30 s, 55°C for 30 s and 72°C for 1 min, with a final extension at 72°C for 5 min. This generated a 340 bp fragment that was then subjected to digestion with MspI (New England Biolabs, Beverly, MA, USA) according to the manufacturer's instruction. The digested product was visualized on a 2% agarose gel, followed by ethidium bromide staining. The presence of the variant allele (m2) was indicated by bands of 200- and 140-bp, whereas no digestion of the wild-type allele (m1) occurred. A modified method, originally described by Oyama and co-workers, was used to identify the other CYP1A1 polymorphism resulting in the substitution of isoleucine for valine at residue 462 in the haem-binding region (Oyama et al, 1995). Genomic DNA was amplified by polymerase chain reaction (PCR) using the primers 5′-GAACTGCCACTTCAGCTGTCT-3′ and 5′-GAAAGACCTCCCAGCGGTCA-3′. The amplification reaction was at 94°C for 5 min to effect initial denaturation, followed by 30 cycles of 20 s at 94°C, 20 s at 55°C, and 40 s at 72°C, with a final extension at 72°C for 5 min. Following amplification, PCR products were digested with HincII (New England Biolabs, Beverly, MA, USA) at 37°C for 3 h. The digested products were electrophoresed on a 3% agarose gel and visualized by ethidium bromide staining. PCR products of Val polymorphism can be cut between codon 462 (GTT) and codon 463 (GAC) by HincII. The GSTM1 genotyping for gene deletion was carried out by PCR amplification with primers for exons 6 and 7 (Chen et al, 1996). All assays were conducted and interpreted blindly to case-control status.

The differences in the allele frequencies of the CYP1A1 gene between HBsAg carriers and non-carriers were tested by multiple linear regression with simultaneous control for ethnic groups and

© Cancer Research Campaign 1999

age. Odds ratio (OR) and confidence interval (CI) were calculated by unconditional logistic regression and adjusted for potential confounding factors. All *P*-values were calculated from two-sided statistical tests.

### RESULTS

### Distributions of CYP1A1 polymorphisms in HBsAg-positive and HBsAg-negative controls

Ninety-eight percent of the study subjects reported that their father and mother had the same ethnic group. Table 1 shows the distribution of the CYP1A1 genetic polymorphisms in HBsAg-positive and HBsAg-negative control subjects by ethnic group. The ethnicity distribution presented was based on fathers' ethnic groups of the study subjects. The MspI variant allele (m2) was less frequent in HBsAg-positive controls than in HBsAg-negative controls, irrespective of ethnic group. Since the Ile-Val polymorphism is closely linked to the MspI polymorphism (Nakachi et al, 1993), the Ile-Val variant allele (Val) was also reduced in frequency among HBsAg carriers in each ethnic group. The overall frequencies of the MspI ($P$ = 0.0377) and Ile-Val ($P$ = 0.0381) variant alleles were significantly lower in HBsAg carriers than in non-carriers after adjustment for ethnic group and age. Since only three HCC cases with available data on CYP1A1 and GSTM1 genotypes were HBsAg-negative, the only way to control for the effect of HBsAg carrier status in stratified analyses was to restrict the analyses to chronic HBV carriers. The following analyses of CYP1A1 polymorphisms and HCC risk were thus carried out among chronic HBV carriers.

### CYP1A1 polymorphisms and HCC risk in chronic HBV carriers

The mean ± standard deviation (s.d.) of ages for the 81 HBsAg-positive HCC cases and 409 HBsAg-positive control subjects in the nested case-control study on CYP1A1 polymorphisms and HCC were 53.1 ± 9.4 and 51.9 ± 9.4 years respectively. Table 2 shows the frequencies of CYP1A1 genotypic variants among HBsAg-positive HCC cases and controls. The MspI allele frequency for the m2 variant type was 43.2% in cases and 39.2% in controls. The corresponding figures for the Ile-Val variant allele were 25.3% and 23.2% respectively. Both MspI and Ile-Val alleles were found to be in Hardy–Weinberg equilibrium. There were no statistically significant differences in the distributions of CYP1A1 polymorphisms between cases and controls.

### Interaction between cigarette smoking and CYP1A1 polymorphism in HCC

After stratifying by status of cigarette smoking, we further examined the associations between CYP1A1 polymorphisms and HCC risk among chronic HBV carriers (Table 3). Among non-smokers, there were essentially no differences in the distributions of CYP1A1 polymorphisms between cases and controls. Conversely, among smokers, the presence of the MspI variant allele significantly increased HCC risk, showing a multivariate-adjusted OR of 3.15 (95% CI 1.20–8.28; $P$ = 0.0196). Cigarette smokers with the Ile-Val variant allele were also at increased risk of HCC (adjusted

**Table 1** Variant CYP1A1 allele frequencies in controls by HBsAg status and ethnic group

| Ethnic group | HBsAg status | Marker (variant allele) MspI (m2) % | Marker (variant allele) Ile-Val (Val) % | Total chromosomes tested |
|---|---|---|---|---|
| Fukien Taiwanese | Negative | 43.2 | 28.8 | 132 |
| | Positive | 39.8 | 23.0 | 470 |
| Hakka Taiwanese | Negative | 52.6 | 36.8 | 38 |
| | Positive | 39.4 | 22.3 | 94 |
| Mainland Chinese | Negative | 47.1 | 27.5 | 138 |
| | Positive | 38.2 | 24.0 | 254 |

**Table 2** Frequency distributions of CYP1A1 genotypes in HBsAg-positive HCC cases and controls

| Genotypes | Cases No. (%) | Controls No. (%) | Adjusted[a] odds ratio (95% CI) |
|---|---|---|---|
| MspI polymorphism | | | |
| m1/m1 | 25 (30.9) | 152 (37.2) | 1.0 |
| m1/m2 | 42 (51.8) | 193 (47.2) | 1.41 (0.81–2.46) |
| m2/m2 | 14 (17.3) | 64 (15.6) | 1.47 (0.71–3.08) |
| Ile-Val polymorphism | | | |
| Ile/Ile | 46 (56.8) | 239 (58.4) | 1.0 |
| Ile/Val | 29 (35.8) | 150 (36.7) | 1.02 (0.60–1.73) |
| Val/Val | 6 (7.4) | 20 (4.9) | 1.61 (0.59–4.38) |

[a]Adjusted for effects of age, cigarette smoking, alcohol drinking, ethnic groups and chronic liver disease

**Table 3** CYP1A1 genetic polymorphisms and HCC risk in HBsAg carriers by status of cigarette smoking

| Genotype | Non-smokers Cases | Non-smokers Controls | Non-smokers OR[a] (95% CI) | Smokers Cases | Smokers Controls | Smokers OR (95% CI) |
|---|---|---|---|---|---|---|
| MspI polymorphism | | | | | | |
| m1/m1 | 19 | 86 | 1.0 | 6 | 66 | 1.0 |
| m1/m2 and m2/m2 | 29 | 158 | 0.92 (0.47–1.78) | 27 | 99 | 3.15 (1.20–8.28)[b] |
| Ile-Val polymorphism | | | | | | |
| Ile/Ile | 33 | 147 | 1.0 | 13 | 92 | 1.0 |
| Ile/Val and Val/Val | 15 | 97 | 0.74 (0.37–1.46) | 20 | 73 | 1.99 (0.91–4.37)[c] |

[a] Adjusted for age, alcohol drinking, ethnic groups and chronic liver disease. [b] $P$ = 0.0196. [c] $P$ = 0.0855.

 © Cancer Research Campaign 1999

**Table 4** Adjusted odds ratios[a] of HCC associated with cumulative exposure to cigarette smoke in HBsAg carriers by combined genotypes of CYP1A1 and GSTM1

| CYP1A1 | GSTM1 | No. of cases | No. of controls[b] | Pack-years of smoking ≤ 16.3 vs 0 | Pack-years of smoking > 16.3 vs 0 |
|---|---|---|---|---|---|
| m1/m1 | Non-null | 12 | 73 | 0.46 (0.05–4.28) | 0.59 (0.11–3.33) |
| m1/m1 | Null | 13 | 77 | 0.29 (0.03–2.47) | 0.66 (0.10–4.53) |
| m1/m2 and m2/m2 | Non-null | 28 | 109 | 0.71 (0.20–2.51) | 1.52 (0.51–4.54) |
| m1/m2 and m2/m2 | Null | 28 | 147 | 3.34 (1.16–9.65)[c] | 1.54 (0.48–4.97) |
| Ile/Ile | Non-null | 24 | 113 | 0.52 (0.10–2.59) | 0.81 (0.25–2.64) |
| Ile/Ile | Null | 22 | 124 | 0.63 (0.16–2.42) | 0.48 (0.11–2.19) |
| Ile/Val and Val/Val | Non-null | 16 | 69 | 0.90 (0.20–4.16) | 1.45 (0.36–5.83) |
| Ile/Val and Val/Val | Null | 19 | 100 | 9.04 (1.92–42.67)[d] | 4.71 (0.94–23.69)[e] |

[a] Adjusted for effects of age, alcohol drinking, and chronic liver disease. [b] One control had no available data on quantity of smoking per day and two controls had no available data on GSTM1 genotype. [c] $P = 0.0259$. [d] $P = 0.0054$. [e] $P = 0.0601$.

**Table 5** HCC risk associated with CYP1A1 MspI polymorphism in HBsAg carriers homozygous for the Ile/Ile genotype of the CYP1A1 Ile-Val polymorphism

| Genotype | Non-smokers Cases | Non-smokers Controls | Non-smokers OR (95% CI) | Smokers Cases | Smokers Controls | Smokers OR (95% CI) |
|---|---|---|---|---|---|---|
| m1/m1 | 19 | 85 | 1.0 | 6 | 64 | 1.0 |
| m1/m2 | 13 | 57 | 1.02 (0.47–2.23) | 5 | 26 | 2.05 (0.58–7.32) |
| m2/m2 | 1 | 5 | 0.89 (0.10–8.11) | 2 | 2 | 10.67 (1.27–89.86)[a] |

[a] $P = 0.0295$.

OR 1.99, 95% CI 0.91–4.37) compared with those without the allele; this association was marginally significant ($P = 0.0855$).

No significant association between cumulative exposure to cigarette smoke and HCC was observed when the association was examined in strata of CYP1A1 genotypes. Because activated metabolites of tobacco-derived PAHs are subjected in part to metabolic detoxification by GSTM1 (Mannervik and Danielson, 1988), the association between pack-years of cigarette smoking and HCC was assessed in categories simultaneously stratified by genotypes of CYP1A1 and GSTM1 (Table 4). Genetic data for GSTM1 were available for all the 81 HBsAg-positive HCC cases and 407 HBsAg-positive controls. Fifty-one percent of the cases and 55% of the controls were the homozygotes for the GSTM1-null genotype which express no protein. After adjusting for potential confounders, the effect of smoking was observed to be most pronounced in those with at least one MspI (OR [95% CI] for smokers of ≤16.3 and > 16.3 pack-years vs non-smokers, 3.34 [1.16–9.65] and 1.54 [0.48–4.97] respectively) or Ile-Val variant allele (OR [95% CI] for smokers of ≤ 16.3 and > 16.3 pack-years vs non-smokers, 9.04 [1.92–42.67] and 4.71 [0.94–23.69] respectively) of CYP1A1 combined with a deficient genotype of GSTM1; however, a monotonic dose–response relation was not observed.

The MspI and Ile-Val variant alleles of CYP1A1 are in linkage disequilibrium (Nakachi et al, 1993). It is likely that the effect of the MspI polymorphism is essentially ascribed to the Ile-Val polymorphism which was associated with gene inducibility and enzymatic activity (Kawajiri et al, 1993; Crofts et al, 1994). This hypothesis was examined in Table 5. Among smoking chronic HBV carriers homozygous for the wild-type allele of the Ile-Val polymorphism, the presence of the MspI variant allele still increased HCC risk. Adjustment for age, alcohol drinking, ethnic groups and chronic liver disease did not materially change the results. This suggests that MspI and Ile-Val polymorphisms may have different mechanisms for increasing susceptibility to smoking-related HCC.

### DISCUSSION

Our previous case-control study found an excess risk of HCC in first-degree relatives of HCC patients, even after adjustment for chronic HBV carrier status (Chen et al, 1991). Segregation analysis of familial HCC demonstrated an interaction between HBV infection and a major genetic locus (Shen et al, 1991). These epidemiological findings suggest that inherited predisposition may be a component of HCC risk. While the relationships between environmental risk factors and HCC have been actively studied, the understanding of the genetic basis of HCC remains primitive.

Both viral and chemical carcinogens are involved in the human hepatocarcinogenesis. Previous epidemiological studies have demonstrated that aflatoxin exposure and cigarette smoking are important risk factors for HCC (Tsukuma et al, 1990; Chen et al, 1991, 1996; Yu et al, 1991; Yu et al, 1997). Aflatoxins and most genotoxic chemical carcinogens in tobacco smoke are not intrinsically reactive but require metabolic conversion to DNA-binding intermediates (Guengerich and Shimada, 1991). While CYP1A2 is the P450 enzyme principally responsible for the bioactivation of aflatoxin $B_1$ at low substrate concentrations associated with dietary

© Cancer Research Campaign 1999 

exposure (Gallagher et al, 1994), CYP1A1 is involved in the metabolic activation of B(a)P and other PAH constituents of cigarette smoke (Shimada et al, 1992). Polymorphisms associated with enzymatic activity of CYP1A1 may determine susceptibility to smoking-related cancer. The frequencies of the CYP1A1 MspI and Ile-Val variant alleles among Chinese in Taiwan are similar to those described for Japanese, but much higher than those in Caucasians (Nakachi et al, 1993; Sivaraman et al, 1994). In this case-control study nested within a prospective study, we found that the relationship between CYP1A1 polymorphisms and HCC in chronic HBV carriers depended on the status of cigarette smoking. The CYP1A1 MspI polymorphism was significantly related to HCC risk in cigarette smokers, whereas no significant association was evident for those who had never smoked. Because the Val allele of the Ile-Val polymorphism is much less frequent than the m2 allele of the MspI polymorphism and the limited sample size, the increased risk of HCC associated with the presence of the Val allele in smokers only reached the borderline significance level. Electrophilic metabolites of B(a)P and other PAHs activated by CYP1A1 are subjected, in part, to metabolic detoxification by GSTM1 (Mannervik and Danielson, 1988). Perhaps individuals with GSTM1 non-null genotypes in combination with the high-risk gene of CYP1A1 are able to eliminate the activated metabolites of PAHs rapidly and efficiently, and thus tolerate carcinogenic insults. The HCC risk associated with cumulative exposure to cigarette smoke in this study was found to be most pronounced among chronic HBV carriers with at least one variant allele of the CYP1A1 MspI, or Ile-Val polymorphism, who also had homozygous deletion of the GSTM1 gene. These results are compatible with the hypothesis that CYP1A1 polymorphism is an important modulator of the hepatocarcinogenic effect of cigarette smoke among chronic HBV carriers. They also indirectly support a role for PAHs in cigarette smoke that are activated by CYP1A1 in the aetiology of HCC. The frequency of the GSTM1-null genotype is high in human populations, approximately 50%, with a reported range of 30–70% (Rebbeck, 1997). Intervention aimed at reducing cigarette smoking may be important for the prevention of HCC in areas where chronic HBV infection and the high-risk gene of CYP1A1 are common.

The frequencies of many polymorphic genetic markers vary markedly between different populations. One major difficulty that often arises in case-control studies of genetic markers and disease is the choice of an appropriate control group for a sample of cases drawn from a mixed population consisting of different subgroups with diverse distributions of genetic markers. Taiwan is an endemic area of HBV infection with a HBsAg carrier rate of 15–20%. Chronic HBV infection has been demonstrated as the most important determinant of HCC in Taiwan (Beasley, 1988; Chen et al, 1991; Yu et al, 1991; Yu and Chen, 1994). In this study, the CYP1A1 MspI and Ile-Val variant alleles were observed to be less prevalent in HBsAg carriers than in non-carriers. This gene frequency difference was present in each ethnic group and was statistically significant after adjustment for ethnic groups and age when all the three ethnic groups were pooled in the analysis. Since only three HCC cases were negative for HBsAg in this study, we thus restricted the analyses of CYP1A1 polymorphisms and HCC to chronic HBV carriers. Although the mechanism responsible for the discrepancies in the allelic frequencies of CYP1A1 polymorphisms between HBsAg carriers and non-carriers is unclear, this study raises the possibility that the failure to take into account HBsAg carrier status in analysis of genetic markers and HCC may cause biased estimates of association, especially in endemic areas of HBV infection.

The Ile-Val polymorphism of the CYP1A1 gene results in the replacement of Ile by Val at amino acid residue 462 in the haem-binding region. The Val variant allele was shown to be associated with increased gene inducibility and catalytic activity (Kawajiri et al, 1993; Crofts et al, 1994). The MspI polymorphism is located in the 3′ non-coding region of the CYP1A1 gene. Although the MspI polymorphism has been shown to co-segregate with the inducibility phenotype of the CYP1A1 enzyme in a family study, the mechanism for this association remains to be established (Petersen et al, 1991). Since the MspI and Ile-Val variant alleles of CYP1A1 are in linkage disequilibrium (Nakachi et al, 1993), it is likely that the association between the MspI polymorphism and HCC among cigarette smokers observed in this study is essentially ascribed to the Ile-Val polymorphism. In smoking chronic HBV carriers homozygous for the wild-type genotype of the Ile-Val polymorphism, however, we found that the MspI variant allele was still associated with increased risk of HCC. This finding suggests that the MspI and Ile-Val polymorphisms may be acting through different mechanisms for influencing susceptibility to smoking-related HCC.

### ACKNOWLEDGEMENTS

This study was supported by grants DOH86-HR-627 and DOH87-HR-627 from the National Health Research Institutes, Department of Health, Executive Yuan; grant NSC87-2314-B-002-291 from the National Science Council of the Republic of China; and grant ES05116 from the National Institutes of Health, USA.

### REFERENCES

Agundez JAG, Olivera M, Ladero JM, Rodriguez-Lescure A, Ledesma MC, Diaz-Rubio M, Meyer UA and Benitez J (1996) Increased risk for hepatocellular carcinoma in NAT2-slow acetylators and CYP2D6-rapid metabolizers. *Pharmacogenetics* **6**: 501–512

Beasley RP (1988) Hepatitis B virus: the major etiology of hepatocellular carcinoma. *Cancer* **61**: 1942–1956

Chen CJ, Liang KY, Chang AS, Chang YC, Lu SN, Liaw YF, Chang WY, Sheen MC and Lin TM (1991) Effects of hepatitis B virus, alcohol drinking, cigarette smoking and familial tendency on hepatocellular carcinoma. *Hepatology* **13**: 398–406

Chen CJ, Yu MW, Liaw YF, Wang LW, Chiamprasert S, Matin F, Hirvonen A, Bell DA and Santella RM (1996) Chronic hepatitis B carriers with null genotypes of glutathione S-transferase M1 and T1 polymorphisms who are exposed to aflatoxin are at increased risk of hepatocellular carcinoma. *Am J Hum Genet* **59**: 128–134

Crofts F, Taioli E, Trachman J, Cosma GN, Currie D, Toniolo P and Garte SJ (1994) Functional significance of different human CYP1A1 genotypes. *Carcinogenesis* **15**: 2961–2963

Gallagher EP, Wienkers LC, Stapleton PL, Kunze KL and Eaton DL (1994) Role of human microsomal and human complementary DNA-expressed cytochrome P4501A2 and P4503A4 in the bioactivation of aflatoxin $B_1$. *Cancer Res* **54**: 101–108

Guengerich FP (1991) Interindividual variation in biotransformation of carcinogens: basis and relevance. In *Molecular Dosimetry and Human Cancer: Analytical, Epidemiological, and Social Considerations*, Groopman JD and Skipper PL (eds), pp. 27–51. CRC Press: Boston

Guengerich FP and Shimada T (1991) Oxidation of toxic and carcinogenic chemicals by human cytochrome P450 enzymes. *Chem Res Toxicol* **4**: 391–407

Hein DW, Doll MA, Rustan TD, Gray K, Feng Y, Ferguson RJ and Grant DM (1993) Metabolic activation and deactivation of arylamine carcinogens by recombinant human NAT1 and polymorphic NAT2 acetyltransferases. *Carcinogenesis* **14**: 1633–1638

 © Cancer Research Campaign 1999

International Agency for Research on Cancer (1986) *IARC Monographs on the Evaluation of the Carcinogenic Risk of Chemicals to Humans. Vol 38, Tobacco Smoking*. IARC: Lyon

Kawajiri K, Nakachi K, Imai K, Watanabe J and Hayashi S (1993) The CYP1A1 gene and cancer susceptibility. *Crit Rev Oncol Hematol* **14**: 77–87

Mannervik B and Danielson UH (1988) Glutathione transferase: structure and catalytic activity. *Crit Rev Biochem Mol Biol* **23**: 281–337

McKinnon RA, Hall PM, Quattrochi LC, Tukey RH and McManus ME (1991) Localization of CYP1A1 and CYP1A2 messenger RNA in normal human liver and in hepatocellular carcinoma by in situ hybridization. *Hepatology* **14**: 848–856

Muscat JE, Richie JP, Jr, Thompson S and Wynder EL (1996) Gender differences in smoking and risk for oral cancer. *Cancer Res* **56**: 5192–5197

Nakachi K, Imai K, Hayashi S and Kawajiri K (1993) Polymorphisms of the CYP1A1 and glutathione S-transferase genes associated with susceptibility to lung cancer in relation to cigarette dose in a Japanese population. *Cancer Res* **53**: 2994–2999

Oyama T, Mitsudomi T, Kawamoto T, Ogami A, Osaki T, Kodama Y and Yasumoto K (1995) Detection of CYP1A1 gene polymorphism using designed RFLP and distributions of CYP1A1 genotypes in Japanese. *In Arch Occup Environ Health* **67**: 253–256

Petersen DD, McKinney CE, Lkeya K, Smith HH, Bale AE, McBride OW and Nebert DW (1991) Human CYP1A1 gene: cosegregation of the enzyme inducibility phenotype and an RFLP. *Am J Hum Genet* **48**: 720–725

Rebbeck TR (1997) Molecular epidemiology of the human glutathione S-transferase genotypes GSTM1 and GSTT1 in cancer susceptibility. *Cancer Epidemiol Biomarkers Prev* **6**: 733–743

Schweikl H, Taylor JA, Kitareewan S, Linko P, Nagorney D and Goldstein JA (1993) Expression of CYP1A1 and CYP1A2 genes in human liver. *Pharmacogenetics* **3**: 239–249

Shen FM, Lee MK, Gong HM, Cai XQ and King MC (1991) Complex segregation analysis of primary hepatocellular carcinoma in Chinese families: interaction of inherited susceptibility and hepatitis B viral infection. *Am J Hum Genet* **49**: 88–93

Shimada T, Yun CH, Yamazaki H, Gautier JC, Beaune PH and Guengerich FP (1992) Characterization of human lung microsomal cytochrome P450 1A1 and its role in oxidation of chemical carcinogens. *Mol Pharmacol* **41**: 856–864

Sivaraman L, Leatham MP, Yee J, Wilkens LR, Lau AF and Marchand LL (1994) CYP1A1 genetic polymorphisms and in situ colorectal cancer. *Cancer Res* **54**: 3692–3695

Spitz MR, Fueger JJ, Beddingfield NA, Annegers JF, Hsu TC and Newell GR (1989) Chromosome sensitivity to bleomycin-induced mutagenesis, an independent risk factor for upper aerodigestive tract cancers. *Cancer Res* **49**: 4626–4628

Talaska G, Al-Juburi AZSS and Kadlubar FF (1991) Smoking related carcinogen-DNA adducts in biopsy samples of human urinary bladder: identification of N-(deoxyguanosin-8-yl)-4-aminobiphenyl as a major adduct. *Proc Natl Acad Sci USA* **88**: 5350–5354

Tiollais P, Pourcel C and Dejean A (1985) The hepatitis B virus. *Nature* **317**: 489–495

Tsukuma H, Hiyama T, Oshima A, Sobue T, Fujimoto I, Kasugai H, Kojima J, Sasaki Y, Imaoka S, Horiuchi N and Okuda S (1990) A case-control study of hepatocellular carcinoma in Osaka, Japan. *Int J Cancer* **45**: 231–236

Wynder EL and Hoffmann D (1994) Smoking and lung cancer: scientific challenges and opportunities. *Cancer Res* **54**: 5284–5295

Yu MC, Tong MJ, Govindarajan S and Henderson BE (1991) Nonviral risk factors for hepatocellular carcinoma in a low-risk population, the Non-Asians of Los Angeles County, California. *J Natl Cancer Inst* **83**: 1820–1826

Yu MW, You SL, Chang AS, Lu SN, Liaw YF and Chen CJ (1991) Association between hepatitis C virus antibodies and hepatocellular carcinoma in Taiwan. *Cancer Res* **51**: 5621–5625

Yu MW and Chen CJ (1994) Hepatitis B and C viruses in the development of hepatocellular carcinoma. *Crit Rev Oncol Hematol* **17**: 71–91

Yu MW, Gladek-Yarborough A, Chiamprasert S, Santella RM, Liaw YF and Chen CJ (1995*a*) Cytochrome P450 2E1 and glutathione S-transferase M1 polymorphisms and susceptibility to hepatocellular carcinoma. *Gastroenterology* **109**: 1266–1273

Yu MW, Hsieh HH, Pan WH, Yang CS and Chen CJ (1995*b*) Vegetable consumption, serum retinol level, and risk of hepatocellular carcinoma. *Cancer Res* **55**: 1301–1305

Yu MW, Lien JP, Santella RM, Liaw YF and Chen CJ (1997) Effect of aflatoxin metabolism and DNA adduct formation on hepatocellular carcinoma among chronic hepatitis B carriers in Taiwan. *J Hepatol* **27**: 320–330

*© Cancer Research Campaign 1999*
